# 685 Patient Satisfaction with Deep Fractional Laser Treatment for Burn Scars in an Outpatient Setting

**DOI:** 10.1093/jbcr/iraf019.314

**Published:** 2025-04-01

**Authors:** Cole Bird, Manal Hassan, Jacob Cindrich, Dhaval Bhavsar

**Affiliations:** University of Kansas School of Medicine; University of Kansas; University of Michigan; The University of Kansas Health System Burnett Burn Center

## Abstract

**Introduction:**

An estimated 486,000 burn injuries requiring medical treatment occur each year, often leading to severe morbidity due to scarring. CO2 laser treatments have become essential for addressing hypertrophic burn scars (HBS). CO2 fractional ablative laser (FAL) treatments usually require use of sedation due to significant pain involved with the procedure. We are using Er:YSGG FAL in select patients in office setting. This study provides outcomes for use of office based burn hypertrophic scar remodeling using Er:YSGG FAL.

**Methods:**

Patients with symptomatic HBS were offered YSGG FAS treatment. Treatments were performed under topical or local anesthesia in office using 240 to 320 mJ energy per square cm and 66 spots per square cm. Multiple treatments were offered with 4-8 weeks between treatment sessions. Treated areas were covered with petroleum-based ointment and healed within 3-5 days. Patients were asked treatment and scar related questions on phone by a non-burn team member. The questionnaire assessed various scar characteristics, including color, stiffness, thickness, irregularity, pain, and itching. Independent t-tests were used to compare the mean scores of Likert scale responses before and after treatment for each scar characteristic.

**Results:**

Patients reported significant improvements after treatment in all assessed scar characteristics: color (3.74 vs. 2.65, p = 0.008), stiffness (3.13 vs. 2.08, p = 0.01), thickness (3.92 vs. 2.08, p < 0.001), irregularity (2.38 vs. 3.58, p = 0.004), pain (2.33 vs. 1.33, p = 0.009), and itching (2.42 vs. 1.54, p = 0.38). Many patients also indicated a reduction in pain and discomfort related to their scars and increased quality of life.

**Conclusions:**

Er:YSGG FAL therapy in an outpatient setting offers a less invasive and highly effective treatment option for select hypertrophic burn scars with smaller surface areas. It provided significant improvements in scar characteristics such as color, stiffness, thickness, and irregularity. Patients also experienced reduced pain and discomfort, thus enhancing quality of life. These findings suggest that office based Er:YSGG FAL therapy may be a viable alternative to CO2 laser treatments, potentially reducing the need for sedation and offering a more accessible, office-based solution for scar management. Future studies with larger cohorts and longer follow-up periods are warranted to further validate these outcomes and explore the long-term benefits of this treatment modality.

**Applicability of Research to Practice:**

Our findings indicate that deep fractional laser therapy, specifically Er:YSGG, can be effectively used in outpatient settings to treat hypertrophic burn scars. This less invasive method shows improvements in scar characteristics and patient satisfaction, offering clinicians a viable alternative to traditional CO2 laser treatments, potentially reducing sedation needs and enhancing access to effective scar management.

**Funding for the Study:**

N/A

